# Lugol’s Lectures on Scrofula

**Published:** 1842-04

**Authors:** 


*Lugol's Lecture on Scrofula.*—The London Medical Gazette for October, contains reports of three lectures on scrofula, by Lugol, of St. Louis Hospital, Paris. These lectures present a very good view of the present state of knowledge on this important subject, and we believe that our readers will be pleased to possess an abstract of them. After noting the importance of the subject, from the frequency of scrofulous disease, Lugol remarks, "that so deeply does scrofula modify the whole organization, that persons afflicted with it may be considered as a *variety of the human race.* Not only is scrofula transmitted by scrofulous parents, but it will often affect all the children of a particular family when the parents fall into a particular state of health (not scrofulous); scrofula though presenting many varieties, is yet *one disease.* M. Lugol recognizes 5 forms—1st, tubercular—2nd, catarrhal—3d, cutaneous—4th, cellular—5th, osseous. Sometimes these forms appear separate; more frequently they are accumulated in the same person, two ore more of them.

1st. *Tubercular Scrofula.*—This is undoubtedly the most important form of scrofula; tubercles co-exist with all other forms of scrofula, they are the emblem of the disease—they are scrofula.

Tubercles are found in every region of the body, in every elementary tissue, in every organ, but wherever found, their anatomical and physical characteristics are the same.

*Form.*—They are round or ovoid, this is the primitive form, but it is often modified by their agglomeration; when not impeded in their development they always assume this form.

*Volume.*—In their origin very small, and even when fully developed not going beyond certain limits. These vary in different organs; in the lungs they never exceed the size of a small pea; in the spleen they are larger, often the size of a bean. In the liver they are larger still, the size of a large olive; this is generally speaking the utmost size that tubercles attain; when they appear larger, it is often because several are agglutinated together. When situated in a region abounding in cellular matter, tubercles sometimes attain the size of an egg or an apricot. In parenchymatous organs they never attain this size.

*Color.*—Generally whitish grey, tinged with yellow, resembling the kernel of a horse.chestnut. The yellow becomes more decided as the tubercle softens. Modifications of the color are very rare. M. L. has seen them of a bright yellow; (qr. from *Jaundice?*) he has seen, though rarely, a mixture of tubercle and melanic matter.

*Density.*—This varies with the period of development; when of a size to be appreciated by the naked eye, they are hard and solid, forming the granules of Laennec and Bayle. Lugol thinks that this is not their first state, but that they follow the general law of organized bodies, and are fluid before they are solid.

*Anatomical Structure.*—Tubercles consist of a cyst or capsule, and a contained matter—tubercular matter. Anatomists have denied their vascularity and attempted to explain their growth by the juxtaposition of tubercnlar matter, secreted by the tissue in which the tubercle is lodged. Lugol thinks that this explanation does not account for the regular growth of the tubercle, and he also claims to have demonstrated the vascularity of these bodies; he says, that "when congestion or inflammation exists, their vascularity is often quite manifest; not only so, but their congestion may lead to rupture of the vessels, and effusion of blood in the substance of the tubercle," of which he communicated a very striking case, in 1828, to the Acad. des Sciences.

*Tubercular Tumors.*—These are constituted of the aggregation of many tubercles—sometimes the line of demarkation can be clearly made out on incising the tumor—sometimes it is quite lost, the mass appearing homogenous, and then again we meet with cases in which the wall of original separation gradually becomes less and less distinct, till finally it disappears altogether.

*Seat of Tubercles.*—Many pathologists insist that tubercles are always developed in lymphatic ganglia, while many, on the contrary, assert that tubercles are in fact only lymphatic glands, which have undergone the tuberculous degeneration. Some have even gone further and called tubercle *glanditis*, thus asserting the inflammatory nature of a disease which only developes itself in persons of debilitated constitutions, and in which antiphlogistic means are most absolutely contra-indicated. Tubercles do not originate in any tissue or in any organ exclusively, nor are they the product of degeneration; every tissue is capable of becoming the matrix in which tubercle may be developed by the agency of those general and hitherto unknown laws to which their production is owing.

Once in being, tubercles go through different periods of development, like all other accidental tissues. Although found in every organ and in every tissue, tubercles are most frequent in parts abounding with cellular tissue, and in lymphatic glands; yet they are found in such parts as the brain, spinal cord, &c., in which no lymphatics have ever been demonstrated. It is in the cervical and inguinal regions, and in the thoracic and abdominal cavities, that tubercles are most common.

*Sub-cutaneous Tubercles.*—These are the most striking manifestations of the tubercular diathesis. In the cervical region, sub-cutaneous tubercles have long borne the name of scrofula or kings-evil; indeed with the older pathologists, scrofula meant only the cervical manifestation of tuberculization.—Even in our own day different names are given to tubercles, according to the region or organ in which they appear: mesenteric tubercles are tabes mesenterica—pulmonary tubercles, phthisis—tubercles beneath the skin are often called tumors. These have all been studied apart, and not as they should be, as mere chapters in the history of tubercular scrofula. Cervical tubercles scarcely ever exist alone, nearly always, not to say always, they are accompanied by tubercles in some region into which the eye cannot penetrate. They are usually small in very young children, and often stationary for a long time; about the end of the first or second septennary period, they increase in size and give rise to tubercular tumors. These may exist on one or both sides, but they are rarely equally developed on both sides. A remarkable fact is that these tumors, however large, do not interfere mechanically with the organs in the cervical regions. Mastication, respiration and deglutition are rarely affected, even by very large tuberculous tumors; this is importont in diagnosticating them. Cervical tubercles are often suspended in branches from the mastoid process, sometimes they extend from that point to the chin, like a collar; they may occupy the sub-clavian hollow, descending beneath the clavicle, and forming a chain with the axillary or mediastinal tubercles. Sometimes they can be traced along the course of nerves or vessels, with whose action they rarely interfere. These remarks equally apply to axillary and inguinal tubercles, which often co-exist with tubercles in other parts. Tubercles are found beneath the skin and sometimes extirpated, being mistaken for sebaceous tumors. This is always useless and often injurious.

*Development of Tubercles in the Splanchnic cavities.*—Tubercles are more frequently seen in the posterior than in the anterior mediastinum, and when they occupy both places, those posterior are largest and most developed. Their presence here generally coincides with their existence elsewhere. They rarely interfere with respiration or deglutition, even when the trachea or bronchia are surrounded by them. Now and then they contract intimate adhesion with the lungs and when they soften and suppurate, ulceration of the pulmonary tissue may ensue.

Tubercles are exceedingly frequent in the peritoneal region in children. In the adult, though frequent, they are rarely accompanied by those symptoms of marasmus, by which their existence in the child is attended. The presence of numerous tubercles in the mesentery is perfectly compatible with good general health. Mesenteric tubercles whether more or less developed, are nearly always perfectly distinct from the lymphatic vessels and ganglions; this explains the compatibility of their existence with a good state of general nutrition. They acquire here a very large size, larger than any where else. What is remarkable, they rarely cause ulceration of the parietes of the intestinal canal, the cœcum nearly alone seems capable of contracting adhesions with them; this is owing to the mobility of the other parts of the intestinal canal; the intestines get out of the way of the tumor as it grows.—*N. Y. Med. Gaz.*

